# Preparation and In Vitro Evaluation of Controlled-Release Matrices of Losartan Potassium Using Ethocel Grade 10 and Carbopol 934P NF as Rate-Controlling Polymers

**DOI:** 10.3390/polym14152993

**Published:** 2022-07-24

**Authors:** Kamran Ahmad Khan, Claudia Zizzadoro, Alessandro Di Cerbo, Nicola Pugliese, Gul Majid Khan, Shakira Ghazanfar, Eman M. Almusalami, Muhammad Muzammal, Khaled J. Alsalman, Arshad Farid

**Affiliations:** 1 Gomal Center of Pharmaceutical Sciences, Faculty of Pharmacy, Gomal University, Dera Ismail Khan 29050, Pakistan; dr.kamran.gu@gmail.com; 2 Department of Veterinary Medicine, University of Bari, 70010 Valenzano, Italy; claudia.zizzadoro@uniba.it; 3 School of Biosciences and Veterinary Medicine, University of Camerino, Via Circonvallazione 93/95, 62024 Matelica, Italy; alessandro.dicerbo@unicam.it; 4 Department of Pharmacy, Quaid-i-Azam University, Islamabad 45320, Pakistan; drgulmajeed@yahoo.com; 5 National Agricultural Research Centre, National Institute of Genomics and Advanced Biotechnology (NIGAB), Park Road, Islamabad 45500, Pakistan; shakira_akmal@yahoo.com; 6 King’s College London, Strand, London WC2R 2LS, UK; eman.al_musalami@kcl.ac.uk; 7 Gomal Center of Biochemistry and Biotechnology, Gomal University, Dera Ismail Khan 29050, Pakistan; mustafamuzammal1@yahoo.com; 8 Pharmaceutical Care Department, Albatha General Hospital, Alodaid 36636, Saudi Arabia; kjalsalman@moh.gov.sa

**Keywords:** formulation, Ethocel grade 10, Carbopol 934P NF, dissolution, kinetic models

## Abstract

Controlled-release formulations are essential for those drugs that require fine tuning of their activity to increase the ratio between therapeutic vs. adverse effects. Losartan potassium is among those drugs whose adverse effects may somehow impair its purported benefits. Previous investigations have been carried out to ascertain the suitability of several polymers for being associated with losartan. This study is focused on the effects of Ethocel grade 10 and Carbopol 934P NF on losartan release. Flow and physical properties were assessed according to the protocols standardized by the pharmacopeia (USP-NF 29), and the drug release in phosphate buffer (pH = 6.8) was measured for 24 h. Data evidenced good to excellent flow and physical properties according to the drug/polymer ratio and the addition of co-excipients. The release rate in 24 h was found to be 63–69% to 79–82% without or with the addition of co-excipients, respectively, following zero-order kinetics. The results also suggest a significant difference with the release profile of a traditional release losartan formulation. The results suggest the suitability of Ethocel grade 10 and Carbopol 934P NF as components of a controlled-release losartan formulation.

## 1. Introduction

The oral route for drug delivery is usually the most suitable one because it is easy to administer, cost-effective concerning dosage development, and safe [1]. However, it may face problems such as fluctuations in plasma levels of the drug, the necessity for repeated administration, and potential side effects [2]. On the other hand, controlled-release (CR) forms overcome those problems, since they increase effectiveness by providing prolonged delivery of the appropriate amount of drug to specific sites for defined periods [3]. For those reasons, several hydrophilic or hydrophobic polymeric matrices have been developed so far. Those matrices allow the control of the drug release by modulating factors such as diffusion, dissolution, and permeation [4].

Controlled-release matrices have been applied to almost all classes of drugs, from antibiotics [5] to anti-cancer compounds [6]. Controlled-release formulations are pivotal for antihypertensive drugs [7] and, among those, several studies have been recently centered on sustained-release forms of losartan, often considered as a model drug for release or solubility investigations [8]. Losartan potassium belongs to the group of angiotensin II receptor blockers, it is freely soluble in water and alcohols, and it is slightly soluble in organic solvents such as acetonitrile. Among clinical uses of losartan potassium, there is the control of high blood pressure or end-organ protection, i.e., in the case of diabetic kidney disease, heart failure, or left ventricular enlargement [9]. It is well known that sustained-release forms of losartan potassium may help in reducing side effects such as migraine, pancreatitis, or hepatotoxicity [8]. Therefore, several losartan potassium sustained-release matrices have been designed, often prepared by the direct compression method by using polymers such as Eudragit RLPO, Eudragit RSPO, and ethylcellulose, either alone or associated with each other, observing an extension of the drug release up to 12 h when using Eudragit polymers in combination with ethylcellulose [10,11], following an anomalous non-Fickian drug release mechanism [11]. Similarly, other authors devised and prepared losartan potassium sustained release matrix tablets by the direct compression method using Kollidon SR [12] and methylcellulose [13] as rate-retarding polymers. Another explored possibility was the preparation of matrix tablets by wet granulation composed of Carbopol 934P and xanthan gum with chitosan, obtaining 99% release of the drug in 24 h [14]. Controlled-release tablets of losartan potassium were also developed by using Ethocel 100 Premium and Ethocel 100 FP Premium, evidencing that the latter extended the drug release rate due to the smaller size of its particle if compared to Ethocel 100 Premium [15]. The incorporation of losartan potassium in a matrix consisting of Ethocel grade 100 and Carbopol was found to further extend the drug release [10].

To widen the panel of further potential matrices, the release rate of a losartan potassium matrix made by Ethocel grade 10 and Carbopol 934P NF was investigated. Additionally, their physical properties were defined. Furthermore, drug release and drug release kinetics were also determined.

## 2. Materials and Methods

### 2.1. Material

Carbopol 934P NF (Lubrizol, Wickliffe, OH, USA), Ethocel grade 10 (Dow Chemical Co., Midland, TX, USA), and losartan potassium (Well & Well Pharma, Islamabad, Pakistan) were purchased from their respective manufacturers. A spectrophotometer (Shimadzu, Japan) was used for the analysis of samples, while a single punch machine (Erweka, Langen, Germany) was used for tablet preparation. A dissolution apparatus (Pharma-Test, Hamburg, Germany) was used for the dissolution study.

All chemicals used in this research study were of analytical grade without any further purification.

### 2.2. Formulation of Tablets

Controlled-release tablets of losartan potassium (Well & Well, Islamabad, Pakistan), were prepared by combining a blend of polymers, namely, Ethocel^®^ grade 10 (granular, hereafter, Ethocel 10P) Premium or Ethocel^®^ grade 10 FP (fine particular, hereafter, Ethocel 10FP) Premium (Dow Chemicals Co., Midland, TX, USA), and Carbopol^®^ 934P (hereafter, Carbopol) NF (Lubrizol, Wickliffe, OH, USA) and losartan potassium with *w*/*w* ratios of 10:3, 10:4, and 10:5, respectively. All tablets contained the same amount of losartan potassium (namely, 50 mg), while the variable component was the polymeric blend. Magnesium stearate 0.5% (Sigma-Aldrich Chemicals Private Limited, Bangalore, India) was added as a lubricant, and spray-dried lactose was used as filler. 

Two further sets of drug-to-polymer ratio (D:P) 10:5 tablets were then prepared by substituting 10% of filler with a correspondent amount of the co-excipients hydroxypropyl methylcellulose (HPMC), carboxymethylcellulose (CMC), or starch. The composition of the tablets is detailed in Table 1. 

### 2.3. Preparation of Matrices

All ingredients were weighed using a digital electronic balance, and drugs and polymers were finely powdered and mixed with the help of a pestle and mortar. After adding excipient (spray dried lactose) and co-excipients (HPMC, CMC, and starch) separately, the mixtures were passed through mesh no. 32 to ensure homogeneous mixing. The lubricant was also mixed and again passed twice through the same mesh to ensure thorough mixing. These mixtures were then compressed into tablets with a single punch tableting machine (Erweka, Langen, Germany). Hardness was maintained within the range of 5–10 kg/cm^2^.

### 2.4. Flow Properties

Flow parameters of the prepared tablets, such as the angle of repose, Carr’s index, Hausner’s ratio, and compressibility index were determined according to the standard procedures [16]. 

### 2.5. Physical Characteristics

The physical characteristics of the matrices were determined according to standardized and well-established procedures. Specifically, the thickness and diameter of 10 randomly selected matrices were measured using a clean Vernier caliper (Erweka, Langen, Germany). The hardness of the other 10 randomly selected matrices was determined by using a hardness tester (Erweka, Langen, Germany). To measure the friability of 20 randomly selected matrices, the latter were placed in a friabilator (Erweka, Langen, Germany) at 100 rpm for 4 min. 

Percent friability was calculated as previously described [10]. Finally, 20 randomly selected matrices were weighed by a digital electronic balance (Shimadzu, Kyoto, Japan). Mean weight and standard deviation (SD) were calculated to verify compliance with the limits reported in the European Pharmacopeia [17].

### 2.6. Chemical Tests

#### 2.6.1. Drug Release Determination

To ascertain the drug release profiles of the CR matrices, USP method-I was employed. Specifically, the experiments were conducted in a dissolution apparatus (Pharma Test Hainburg, Germany). Following preliminary tests, 900 mL of 0.2 M phosphate buffer (pH 6.8) was used as a dissolution medium. During the dissolution experiments, the temperature was kept at 37 ± 0.5 °C, and the rotation of baskets was maintained at 100 rpm. At the beginning of the experiment (T0) and after 0.5, 1, 1.5, 2, 3, 4, 6, 8, 10, 12, 18, and 24 h, 5 mL samples were collected and filtered through 0.45 μm membranes to remove possible particulate matter. The filtered samples were analyzed using a spectrophotometer (Shimadzu) at λ = 205 nm. Since Ethocel 10FP and Carbopol are not soluble in water, filtration was needed to remove the potential presence of excipients that could interfere with the spectrophotometric analysis.

A reference solution was prepared by adding 100 mg of losartan potassium to 100 mL of phosphate buffer and dissolved. Serial dilutions were obtained by diluting 1 mL of the reference solution in a 99 mL phosphate buffer. Therefore, the standard curve was obtained by measuring the absorbance of reference solutions in each experiment set. The standard curve was used to calculate the concentration of the samples and, consequently, the drug release by considering the initial amount of drug and the volume of the dissolution medium. Each experiment was performed in triplicate. Conventional tablets (Cardaktin^®^), each containing 100 mg of losartan potassium, were used as a control.

#### 2.6.2. Content Uniformity Determination

Twenty tablets were randomly selected and powdered, and the powder mass corresponding to 100 mg of drug was dissolved in 100 mL of 0.2 M phosphate buffer (pH 6.8). One mL aliquots were then diluted in 99 mL of phosphate buffer. Five mL of the diluted sample solution were filtered to avoid possible particulate material and interference with the spectrophotometric analysis and drug absorbance (λ = 205 nm) was spectrophotometrically determined. Properly diluted reference solutions were used to obtain the standard curve. The drug content uniformity was calculated from absorbances of reference and sample solutions and evaluated according to the current standards [18].

### 2.7. Statistical Analysis

Mean and standard deviation were calculated for each set of flow property tests. The difference among them was checked by one-way ANOVA, and the Tukey HSD post hoc test was performed to verify the pairwise significance. The significance limit was set at *p* < 0.05.

The data obtained from the drug release experiments were verified for their fitness in different mathematical models such as zero-order kinetics, first-order kinetics, Hixon Crowell’s cube-root equation, Higuchi’s square root of time equation, and the Korsmeyer–Peppas model (Power law) [19].

The dissolution rates were compared by applying difference and similarity factors f1 and f2, respectively [10].

## 3. Results

### 3.1. Flow Features and Physical Properties of Matrices

The flow and physical properties of all the matrix sets were found to be acceptable, being within the ranges of “Excellent” or “Good” as defined by the US pharmacopeia [16] (Table 2).

By analyzing the quantitative data from the formulations obtaining excellence in all three parameters, a slight but significant (*p* < 0.01) difference was observed between the angle of repose of formulations S1 and S2 (Ethocel 10P/Carbopol D:P 10:3 and 10:4, respectively, *p* < 0.01), and S2 and S6 (Ethocel 10P/Carbopol D:P 10:4 and Ethocel 10FP/Carbopol D:P 10:5, respectively, *p* < 0.001), while no significant difference was observed between S1 and S6 (Ethocel 10P/Carbopol D:P 10:3 and Ethocel 10FP/Carbopol D:P 10:5, respectively). No significant difference was observed in Hausner’s ratio and Carr’s index among S1, S2, and S6.

When co-excipients were added, all flow properties fell in the excellent range in tablets where starch was added to Ethocel 10P/Carbopol D:P 10:5, but the angle of repose and the Carr’s index of the latter were significantly lower (*p* < 0.001), while no significant difference was observed in the Hausner’s ratio.

Among physical features, the values of which are listed in Table 3, diameter and thickness were found to be constant among the different sets. Limited but significant variability was observed among the experimental sets and for hardness (MS = 0.063, *p* < 0.001), while greater variability was calculated for friability (MS = 0.075, *p* < 0.01).

The difference in weight within tablets of the same set was found to be within the limits indicated by the US pharmacopeia [16]. Similarly, the drug content was found to be uniform in the matrices of the same experimental sets (Table 4), all ranging between 98.52 and 99.48%, within the limits indicated by the US pharmacopeia [18].

### 3.2. Drug Release

**Figure 1:**
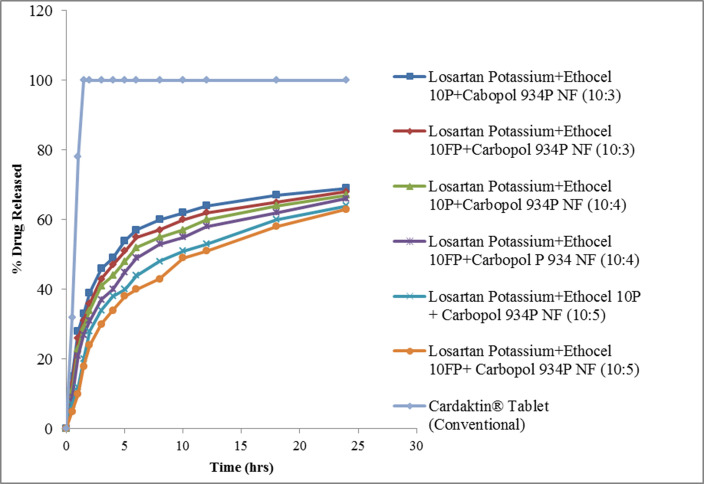
Drug release from polymeric matrices. The points in the curves indicate the mean value of three repetitions.

The release curves of losartan potassium from Ethocel 10P or Ethocel 10FP and Carbopol are shown in Figure 1.

**Figure 2:**
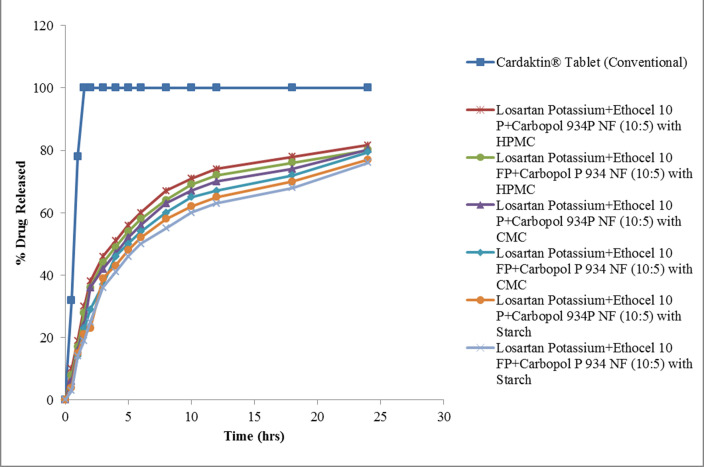
Drug release from polymeric matrices when co-excipients were added. The points in the curves indicate the mean value of three repetitions.

After 24 h, 63% (Ethocel 10 FP/Carbopol D:P 10:5 matrix) to 69% (Ethocel 10P/Carbopol D:P 10:3) of losartan potassium was released, the formulation with the higher D:P being more prone to release the drug (*p* < 0.05). The addition of co-excipients sensibly increased the drug release rate. Specifically, the addition of HPMC led to the release of 82% and 81% of the drug within 24 h in matrices with Ethocel 10P/Carbopol and Ethocel 10FP/Carbopol, respectively, both with D:P 10:5. Starch brought the release rate to 80.33% and 79.43% in D:P 10:5 matrices with Ethocel 10P/Carbopol and Ethocel 10FP/Carbopol, respectively, and, finally, CMC increased the 24 h drug release to 81.62% and 80.05% in D:P 10:5 matrices with Ethocel 10P/Carbopol and Ethocel 10FP/Carbopol, respectively (Figure 2).

The model analyses showed that the highest values of r^2^ were obtained when data were fitted to Higuchi’s model, falling in an interval between 0.973 and 0.995 (Table 5).

Worst fitness parameters were obtained, when trying to fit them, to first-order kinetics (0.476 < r^2^ < 0.886). Interestingly, when fitted with zero-order kinetics, all but one set exhibited r^2^ values higher than 0.98 were obtained. The exception was represented by the experimental set S7 (Ethocel 10P/Carbopol D:P 10:5 with HPMC), the r^2^ for the zero-order kinetics which was 0.765.

Finally, when the adherence to the Power law was assessed, N values ranged from 0.629 to 0.974, indicating an anomalous, non-Fickian diffusion of the drug.

When the dissolution profiles of the experimental matrices were compared with traditional release tablets (namely, Cardaktin^®^), the average difference factor (f_1_) was 49.04 ± 5.41, despite that value being higher than 54 in all but one experimental set without co-excipients (Table 6).

Conversely, the mean f_2_ value was 13.82 ± 2.38, with values of sets S3 and S6 (Ethocel 10P/Carbopol D:P 10:5 and Ethocel 10FP/Carbopol 10:5, respectively) higher than 17.3.

## 4. Discussion

The drug release pattern is a pivotal aspect, especially when applied to formulations for oral administration since it allows for fine temporal control of the drug circulation in the human or animal host, thus tuning its therapeutic effects and preventing some of the potential adverse effects [20]. Polymers are widely recognized as the most effective molecules associated with drugs for preparing controlled-release formulations, and the appropriate polymer selection is crucial for optimizing the drug encapsulation, dissolution, and, consequently, activity [21].

Till now, several polymers have been tested for their effects on losartan potassium release. Among them, Eudragit, ethylcellulose, and Kollidon [10,11,12] were found to be effective when included in sustained-release formulations. Additionally, a recent study assessed the properties of losartan potassium tablets prepared by adding ethylcellulose Ethocel grade 100 and Carbopol 934P NF [10].

The here-presented results showed that losartan potassium tablets prepared with Ethocel grade 10 and Carbopol 934P NF present similar features if compared with those obtained by using Ethocel grade 100. Ethocel 10P and Carbopol greatly enhanced the flow properties of losartan potassium, which were otherwise poor. Interestingly, the best flow performances were obtained when the D:P ratio was 10:5. The use of Ethocel P or FP seems to affect the flow properties since the best results were obtained with Ethocel 10 FP. However, the tablets prepared with Ethocel 10 FP were harder. The addition of co-excipients (HPMC, CMC, or starch) did not improve the flow properties but reduced the friability.

In all cases, the matrices complied with the indications devised by the most common pharmacopeia standards and protocols [16,17,18].

On the other hand, the drug release curves strongly fitted the zero-order kinetics model, which is considered optimal [22], thus suggesting continuous and constant solubilization of the drug. The physical and chemical properties of ethylcellulose and Carbopol may explain such a mechanism, since the latter is a hydrophilic polymer. It swells in alkaline pH, forming hydrogels due to the ionization of its acidic groups and producing repulsion between the negative charges, thus promoting the release of drugs through that gel layer, while ethylcellulose is a retardant matrix, which controls the swelling of the gel [25]. The N values obtained from the study of the Korsmeyer–Peppas model, ranging from 0.5 and ranging between 0.60 and 0.89, with only one exception, suggest an anomalous, non-Fickian release, probably due to a diffusion process hampered by the polymeric gel relaxation [23]. Other than the zero-order kinetics, the drug release curves have also been found to fit Higuchi’s diffusion model, which related the drug diffusion to the square root of time and is valid when polymers of the matrix do not change their properties when in contact with an aqueous medium [19].

However, it should be underlined that less than 70% of the drug is released after 24 h. This is in agreement with the previous findings with Ethocel grade 100, but not if compared to matrices prepared with Eudragit [8] or Kollidon SR [9], which allowed more drugs to be released, despite the release being much less gradual with Kollidon.

It is noteworthy that the release after 24 h improved following the addition of co-excipients, especially HPMC. All of them are water-soluble polymers that enhance the drug release rate [24]. In particular, HPMC is a non-ionic polymer that is rapidly hydrated and may help in releasing the drug by increasing the local osmotic pressure [25].

Despite the increased release rate, the addition of co-excipients did not significantly affect the kinetics of the process, which remained significantly different when compared with Cardaktin^®^ tablets. The f1 and f2 values, measures of the difference and the similarity between two analyzed curves, were mostly or nearly within the relevance intervals, namely, 0–15 for f1 and 50–10 for f2 [26], thus suggesting a significant deviation from the traditional release mechanisms.

## 5. Conclusions

All the above considered, the present study suggests that the controlled-release matrices developed by blending Ethocel grade 10 and Carbopol 934P NF showed good results in terms of physicochemical parameters and drug release profiles. The addition of co-excipients such as HPM, CMC, and starch may help in increasing the release rate, and further study might be undertaken to develop optimal concentrations to obtain the desired release rate, therefore achieving the important goal of finely tuning the pharmaceutical cargo to tailor the therapy to the needs of the patients.

## Figures and Tables

**Table 1:** Tablet composition.

Set	D:P	Losartan Potassium (%)	Polymers	Polymers Rate (%)	Magnesium Stearate (%)	Spray-Dried Lactose (%)	Co-Excipient *
S1	10:3	50	Ethocel 10 Premium Carbopol 934P NF	15	0.5	34.5	None
S2	10:4	50	Ethocel 10 Premium Carbopol 934P NF	20	0.5	29.5	None
S3	10:5	50	Ethocel 10 Premium Carbopol 934P NF	25	0.5	24.5	None
S4	10:3	50	Ethocel 10 FP Premium Carbopol 934P NF	15	0.5	34.5	None
S5	10:4	50	Ethocel 10 FP Premium Carbopol 934P NF	20	0.5	29.5	None
S6	10:5	50	Ethocel 10 FP Premium Carbopol 934P NF	25	0.5	24.5	None
S7	10:5	50	Ethocel 10 Premium Carbopol 934P NF	25	0.5	22.05	HPMC
S8	10:5	50	Ethocel 10 Premium Carbopol 934P NF	25	0.5	22.05	CMC
S9	10:5	50	Ethocel 10 Premium Carbopol 934P NF	25	0.5	22.05	Starch
S10	10:5	50	Ethocel 10 FP Premium Carbopol 934P NF	25	0.5	22.05	HPMC
S11	10:5	50	Ethocel 10 FP Premium Carbopol 934P NF	25	0.5	22.05	CMC
S12	10:5	50	Ethocel 10 FP Premium Carbopol 934P NF	25	0.5	22.05	Starch

* When present, co-excipient represents 10% of the filler. HPMC: hydroxypropyl methylcellulose; CMC: carboxymethylcellulose.

**Table 2:** Flow properties of the tested matrices.

Experimental Set	Formulations	Flowability		Hausner’s Ratio		Compressibility	
		Angle of Repose (Plain Degrees)	Qualitative Character [11]	Hausner’s Ratio	Qualitative Character [11]	Carr’s Index (%)	Qualitative Character [11]
SC	Losartan potassium powder	54.23 ± 0.89	Poor	1.45 ± 0.56	Poor	31.45 ± 0.54	Poor
S1	Ethocel 10P/Carbopol 934P NF (10:3)	25.26 ± 0.15	Excellent	1.01 ± 0.72	Excellent	9.2 ± 0.45	Excellent
S2	Ethocel 10P/Carbopol 934P NF (10:4)	26.43 ± 0.73	Excellent	1.03 ± 0.43	Excellent	9.4 ± 0.25	Excellent
S3	Ethocel 10P/Carbopol 934P NF (10:5)	33.77 ± 0.56	Good	1.17 ± 0.49	Good	12.87 ± 0.32	Good
S4	Ethocel 10FP/Carbopol 934P NF (10:3)	30.38 ± 0.36	Excellent	1.15 ± 0.65	Good	11.52 ± 0.33	Good
S5	Ethocel 10FP/Carbopol 934P NF (10:4)	31.62 ± 0.39	Good	1.15 ± 0.54	Good	13.09 ± 0.65	Good
S6	Ethocel 10FP/Carbopol 934P NF (10:5)	24.86 ± 1.34	Excellent	0.9 ± 0.36	Excellent	8.89 ± 0.76	Excellent
S7	Ethocel 10P/Carbopol 934P NF (10:5) with HPMC	33.72 ± 0.09	Good	1.17 ± 0.38	Good	14.57 ± 0.53	Good
S8	Ethocel 10P/Carbopol 934P NF (10:5) with CMC	28.26 ± 0.27	Excellent	1.08 ± 0.18	Excellent	11.54 ± 0.18	Good
S9	Ethocel 10P/Carbopol 934P NF (10:5) with Starch	29.61 ± 0.55	Excellent	1.10 ± 0.34	Excellent	10.30 ± 0.66	Excellent
S10	Ethocel 10FP/Carbopol 934P NF (10:5) with HPMC	32.34 ± 0.58	Good	1.15 ± 0.83	Good	13.65 ± 0.45	Good
S11	Ethocel 10FP/Carbopol 934P NF (10:5) with CMC	30.45 ± 0.33	Excellent	1.14 ± 0.13	Good	11.90 ± 0.53	Good
S12	Ethocel 10FP/Carbopol 934P NF (10:5) with Starch	30.81 ± 0.34	Good	1.13 ± 0.30	Good	12.47 ± 0.55	Good

**Table 3:** Physical properties of the tested matrices.

Experimental Set	Formulations	Diameter (mm, n = 10)	Thickness (mm, n = 10)	Hardness (kg/cm^2^, n = 10)	Friability (%, n = 20)	Weight Variation (mg, n = 20)
S1	Ethocel 10P + Carbopol 934P NF (10:3)	8.0 ± 0.26	2.5 ± 0.19	8.7 ± 0.11	0.11 ± 0.45	200 ± 0.42
S2	Ethocel 10P + Carbopol 934P NF (10:4)	8.0 ± 0.17	2.5 ± 0.31	8.9 ± 0.15	0.12 ± 0.04	199 ± 0.34
S3	Ethocel 10P + Carbopol 934P NF (10:5)	8.0 ± 0.26	2.5 ± 0.43	8.3 ± 0.33	0.31 ± 0.11	202 ± 0.13
S4	Ethocel 10FP + Carbopol 934P NF (10:3)	8.0 ± 0.38	2.4 ± 0.06	9.7 ± 0.10	0.10 ± 0.18	201 ± 0.22
S5	Ethocel 10FP+ Carbopol 934P NF (10:4)	8.0 ± 0.13	2.4 ± 0.29	9.8 ± 0.05	0.20 ± 0.05	201 ± 0.25
S6	Ethocel 10FP + Carbopol 934P NF (10:5)	8.0 ± 0.33	2.4 ± 0.15	9.5 ± 0.07	0.23 ± 0.09	203 ± 0.44
S7	Ethocel 10P + Carbopol P934 NF (10:5) with HPMC	8.0 ± 0.87	2.5 ± 0.79	8.3 ± 0.25	0.03 ± 0.22	202 ± 0.35
S8	Ethocel 10P + Carbopol P934 NF (10:5) with CMC	8.0 ± 0.43	2.5 ± 0.35	9.3 ± 0.17	0.03 ± 0.16	201 ± 0.04
S9	Ethocel 10P + Carbopol P934 NF (10:5) with Starch	8.0 ± 0.40	2.5 ± 0.23	8.5 ± 0.28	0.01 ± 0.66	203 ± 0.16
S10	Ethocel 10FP + Carbopol P934 NF (10:5) with HPMC	8.0 ± 0.97	2.4 ± 0.53	8.6 ± 0.43	0.06 ± 0.17	200 ± 0.33
S11	Ethocel 10FP + Carbopol P934 NF (10:5) with CMC	8.0 ± 0.26	2.4 ± 0.24	9.5 ± 0.43	0.03 ± 0.25	199 ± 0.29
S12	Ethocel 10FP + Carbopol P934 NF (10:5) with Starch	8.0 ± 0.37	2.4 ± 0.19	9.3 ± 0.43	0.06 ± 0.22	200 ± 0.25

**Table 4:** Content uniformity of the sets of matrices.

Experimental Set	Formulations	Content Uniformity (%, n = 10)
S1	Ethocel 10P + Carbopol 934P NF (10:3)	99.04
S2	Ethocel 10P + Carbopol 934P NF (10:4)	99.42
S3	Ethocel 10P + Carbopol 934P NF (10:5)	98.67
S4	Ethocel 10FP + Carbopol 934P NF (10:3)	99.18
S5	Ethocel 10FP+ Carbopol 934P NF (10:4)	98.88
S6	Ethocel 10FP + Carbopol 934P NF (10:5)	99.02
S7	Ethocel 10P + Carbopol 934P NF (10:5) with HPMC	99.35
S8	Ethocel 10P + Carbopol 934P NF (10:5) with CMC	98.56
S9	Ethocel 10P + Carbopol 934P NF (10:5) with Starch	99.34
S10	Ethocel 10FP + Carbopol 934P NF (10:5) with HPMC	99.22
S11	Ethocel 10FP + Carbopol 934P NF (10:5) with CMC	99.08
S12	Ethocel 10FP + Carbopol 934P NF (10:5) with Starch	98.78

**Table 5:** Drug release kinetics parameters (n = 3, mean ± SD).

Experimental Sets	Formulations	First-Order Kinetic		Zero-Order Kinetic		Hixon Crowell’s Erosion Model		Higuchi Diffusion Model		Power Law		
		k_1_ ± SD	r^2^	k_2_ ± SD	r^2^	k_3_ ± SD	r^2^	k_4_ ± SD	r^2^	k_5_ ± SD	r^2^	N
S1	Ethocel 10P + Carbopol 934P NF (10:3)	−0.337 ± 0.39	0.873	7.323 ± 0.324	0.983	0.342 ± 0.254	0.939	7.633 ± 0.538	0.984	0.003 ± 0.028	0.963	0.676
S2	Ethocel 10P + Carbopol 934P NF (10:4)	−0.323 ± 0.32	0.867	7.747 ± 0.212	0.993	0.283 ± 0.232	0.872	6.721 ± 0.634	0.994	0.005 ± 0.067	0.931	0.629
S3	Ethocel 10P + Carbopol 934P NF (10:5)	−0.368 ± 0.29	0.859	8.334 ± 0.332	0.982	0.236 ± 0.476	0.898	7.732 ± 0.337	0.973	0.026 ± 0.176	0.975	0.974
S4	Ethocel 10FP + Carbopol 934P NF (10:3)	−0.383 ± 0.27	0.862	8.455 ± 0.423	0.989	0.439 ± 0.276	0.895	7.665 ± 0.597	0.990	0.004 ± 0.155	0.936	0.647
S5	Ethocel 10FP + Carbopol 934P NF (10:4)	−0.387 ± 0.42	0.787	7.986 ± 0.654	0.994	0.283 ± 0.233	0.832	6.629 ± 0.543	0.995	0.006 ± 0.338	0.933	0.694
S6	Ethocel 10FP + Carbopol 934P NF (10:5)	−0.161 ± 0.23	0.674	8.789 ± 0.532	0.983	0.257 ± 0.584	0.936	7.797 ± 0.569	0.984	0.082 ± 0.284	0.982	0.887
S7	Ethocel 10P + Carbopol 934P NF (10:5) with HPMC	−0.424 ± 0.42	0.476	4.345 ± 0.228	0.765	0.219 ± 0.185	0.917	4.779 ± 0.225	0.983	0.023 ± 0.065	0.965	0.745
S8	Ethocel 10P + Carbopol 934P NF (10:5) with CMC	−0.33 ± 0.28	0.879	3.364 ± 0.182	0.989	0.281 ± 0.516	0.878	4.451 ± 0.332	0.983	0.013 ± 0.051	0.982	0.787
S9	Ethocel 10P + Carbopol 934P NF (10:5) with Starch	−0.348 ± 0.2	0.876	5.327 ± 0.559	0.988	0.368 ± 0.126	0.985	5.189 ± 0.134	0.984	0.037 ± 0.139	0.956	0.676
S10	Ethocel 10FP + Carbopol 934P NF (10:5) with HPMC	−0.439 ± 0.43	0.867	3.238 ± 0.128	0.986	0.221 ± 0.284	0.969	3.118 ± 0.435	0.988	0.018 ± 0.087	0.949	0.795
S11	Ethocel 10FP + Carbopol 934P NF (10:5) with CMC	−0.23 ± 0.17	0.866	3.389 ± 0.187	0.986	0.118 ± 0.168	0.923	4.863 ± 0.642	0.989	0.034 ± 0.013	0.962	0.779
S12	Ethocel 10FP + Carbopol 934P NF (10:5) with Starch	−0.354 ± 0.3	0.886	4.387 ± 0.355	0.985	0.652 ± 0.764	0.978	5.578 ± 0.348	0.987	0.065 ± 0.068	0.881	0.880

**Table 6:** Drug release comparison between the tested matrices and commercial, traditional release losartan potassium tablets (Cardaktin^®^).

Experimental Set	Formulation	Difference Factor (f_1_)	Similarity Factor (f_2_)
S1	Ethocel 10P + Carbopol 934P NF (10:3)	55.41	12.26
S2	Ethocel 10P + Carbopol 934P NF (10:4)	54.78	13.04
S3	Ethocel 10P + Carbopol 934P NF (10:5)	43.83	18.75
S4	Ethocel 10FP + Carbopol 934P NF (10:3)	55.78	10.45
S5	Ethocel 10FP + Carbopol 934P NF (10:4)	59.31	11.27
S6	Ethocel 10FP + Carbopol 934P NF (10:5)	51.42	17.79
S7	Ethocel 10P + Carbopol 934P NF (10:5) with HPMC	44.38	14.63
S8	Ethocel 10P + Carbopol 934P NF (10:5) with CMC	46.30	13.53
S9	Ethocel 10P + Carbopol 934P NF (10:5) with Starch	43.85	13.91
S10	Ethocel 10FP + Carbopol 934P NF (10:5) with HPMC	47.27	13.46
S11	Ethocel 10FP + Carbopol 934P NF (10:5) with CMC	47.05	13.36
S12	Ethocel 10FP + Carbopol 934P NF (10:5) with Starch	45.43	13.35

## Data Availability

The data presented in this study are available on request from the corresponding author.
